# The research progress in the interaction between *Candida albicans* and cancers

**DOI:** 10.3389/fmicb.2022.988734

**Published:** 2022-09-28

**Authors:** Dalang Yu, Zhiping Liu

**Affiliations:** ^1^School of Basic Medicine, Fuzhou Medical College of Nanchang University, Fuzhou, Jiangxi, China; ^2^School of Basic Medicine, Gannan Medical University, Ganzhou, Jiangxi, China

**Keywords:** *Candida albicans*, cancer, inflammation, interaction, therapy

## Abstract

*Candida albicans* is an opportunistic pathogenic fungus, which tends to infect the host with defective immune function including cancer patients. A growing number of studies have shown that *C. albicans* infection increases the host susceptibility to cancer such as oral, gastric, and colorectal cancer. Cancer and anti-cancer treatment may also affect the colonization of *C. albicans. C. albicans* may promote the development of cancer by damaging mucosal epithelium, inducing the production of carcinogens, triggering chronic inflammation including Th17 cell-mediated immune response. In this article, we aim to elaborate the interaction between *C. albicans* and cancers development and summarize the potential molecular mechanisms, so as to provide theoretical basis for prevention, diagnosis and treatment of cancers.

## Introduction

*Candida* is a group of opportunistic pathogenic fungi, which are often found in the host’s skin, mouth, and gastrointestinal tract. The decrease in the host immunity enhances the risk of *Candida* infection. Among them, *Candida albicans* is the most common pathogenic fungus (Mba and Nweze, 2020). In recent years, more and more epidemiological and pathological studies have suggested the significant impact of pathogenic microorganisms on the incidence rates of cancers worldwide (Schottenfeld and Beebe-Dimmer, 2015). However, only some studies have linked fungal infection with cancers. The association between fungal microbiota imbalance and carcinogenesis remains largely unknown. This is due to the relatively low abundance of fungi and the lack of well-defined reference genome (Klimesova et al., 2018; Coker et al., 2019). In addition, the research methods are challenging, so the fungal microbiota is usually explored relatively less than other microorganisms. So far, studies have shown that *C. albicans* in fungal microorganisms is very closely related to cancer development.

In this article, we aim to review the potential molecular mechanism by which *C. albicans* promotes cancers progression. This may help clinicians diagnose the early stage of tumor in the future and prescribe a treatment method considering the possible microbial properties in the process of carcinogenesis.

## *Candida albicans* infection may promote the development of cancers

In recent years, studies have found that *C. albicans* infection is closely related to cancers. On the one hand, cancer patients with defective immune function have increased risk of fungal infection; on the other hand, fungus infection may affect the occurrence and development of cancer in different ways.

### *Candida albicans* and oral cancer

In the observational study, a link between *Candida* infection induced oral leukoplakia and malignant tumors was found. *Candida* infection occurred in 13.5% of oral leukoplakia cases, and in 28.7% of the malignant tumor cases (Banoczy and Sugar, 1975; Warnakulasuriya and Ariyawardana, 2016; Di Cosola et al., 2021). Another study showed that 31% of 257 patients with oral leukoplakia were infected with *Candida* (Silverman et al., 1984). In the leukoplakia cancer group, 53% of patients were *Candida* positive before tumor formation (Perera et al., 2017). One study identified that *C. albicans* was the most frequently detected and abundant fungus in oral squamous cell carcinoma (OSCC) (Mäkinen et al., 2018). In addition, *C. albicans* was also the most commonly isolated *Candida* species from saliva samples of patients with oral cancer (Jain et al., 2016). From the current epidemiological studies, the detection rates of *Candida* in patients with oral cancer are increased, and *C. albicans* is the main one. Unfortunately, these are only epidemiological and descriptive studies, lacking effective experimental evidence. Therefore, the above studies can only show that *C. albicans* is associated with cancer development, but there is no definite causal relationship.

In order to prove the role of *C. albicans* infection in oral cancer, some researchers have carried out relevant experimental studies. It was found that the virulence attributes of *Candida* and the production capacity of ethanol derived acetaldehyde were related to the development of oral cancer (Alnuaimi et al., 2016). Furthermore, the high biofilm forming ability of *Candida* may ensure the long-term exposure of host tissue to fungal carcinogens such as acetaldehyde, and increased production of hydrolases may trigger chronic inflammatory responses in the host tissues (Alnuaimi et al., 2016). Chronic inflammation caused by microbial infection is one of the risk factors of tumor development (Karin and Greten, 2005). However, above study did not identified the correlation between the severity of inflammatory changes at cancer sites and the virulence of *Candida* isolated from these sites. Another study showed that zymosan from the cell wall of *C. albicans* promoted the proliferation of OSCC cells through TLR2/MyD88/NF-κB signaling pathway. In addition, zymosan could promote the expression of E-cadherin, thus enhancing the adhesion of *C. albicans* onto OSCC cells and further increasing IL-1β production (Chen et al., 2020). Meanwhile, zymosan was shown to participate in the IL-1β secretion by OSCC cells by regulating the NLRP3/IL-1β pathway. However, the secretion of proinflammatory cytokines (such as IL-1β) is also influenced by the microbiota or its cellular components, indicating a more complex interaction between cancer cells, immune cells, and the microbiota in the tumor microenvironment (TME) (Chen et al., 2020). NLRP3 inflammasome-induced IL-1β production promoted 5-FU resistance in OSCC both *in vitro* and *in vivo* (Feng et al., 2017). It can be inferred that the presence of *C. albicans* in oral cancer may influence the effect of chemotherapy by inducing IL-1β production, which was also a potential target for treating oral cancer. Some studies also found that *C. albicans* strains from oral precancerous lesions showed the highest nitrosation potential, while *Candida tropicalis*, *Candida parapsilosis*, and *Torulopsis glabrata* have lower nitrosation potential, which can induce epithelial carcinogenesis and promote tumor development (Krogh, 1990). Another study detected the prevalence of *C. albicans* alcohol dehydrogenase 1 (*CaADH1*) gene in oral dysplasia and OSCC and found that *CaADH1* gene is associated with OSCC either with or without metastasis, indicating that it may be related to the progression and metastasis of OSCC (Hafed et al., 2019). However, they were unable to confirm whether the observed *Candida* infection was related to therapeutic interventions such as surgery or chemotherapy. Therefore, it is uncertain whether the development of OSCC is the primary or secondary effect of *C. albicans* infection. A further *in vitro* study found that the presence of living *C. albicans* promoted the progress of OSCC by stimulating the production of matrix metalloproteinases (MMPs), tumor metabolites, tumor promoting signal pathways. These results suggest that *C. albicans* actively participates in the complicated process of OSCC progression (Vadovics et al., 2022; Table 1). However, the specific roles of *C. albicans* in the development of oral cancer remains unclear.

**TABLE 1:** The association of *C. albicans* and cancers.

Fungus	Associated cancer	Main hypothetical molecular mechanisms	References
*C. albicans*	Oral cancers	Form biofilm, produce hydrolase, and metabolize alcohol into carcinogenic acetaldehyde.	Marttila et al., 2013; Alnuaimi et al., 2015, 2016
		Increases p63 and vimentin expression and decreases E-cadherin expression.	Lo Muzio et al., 2007; Kushwaha et al., 2019; Vadovics et al., 2022
		Zymosan from the fungal cell wall promotes the proliferation of oral squamous cell carcinoma (OSCC) cells through the TLR2/MyD88/NF-κB signaling pathway. Moreover, zymosan can promote the expression of E-cadherin, enhance the adhesion of *C. albicans* to OSCC cells, and further increase IL-1β production in OSCC cells, and promote cancerous inflammation.	Chen et al., 2020
		Produce endogenous nitrosamines.	Krogh, 1990
		*CaADH1* gene is involved in OSCC either with or without metastasis.	Hafed et al., 2019
	Gastric cancer (GC)	Reduction in the diversity and richness of fungi in the stomach contributes to the pathogenesis of GC.	Zhong et al., 2021
	Colorectal cancer (CRC)	Enteric fungal microbiota dysbiosis and ecological alterations. *Candida albicans* increases glycolysis levels in macrophages through the HIF-1 pathway, prompting IL-7 secretion and release from macrophages. The increase of IL-7 effectively promotes the expression level of Stat3 and AhR transcription factors in intestinal innate lymphocytes 3 (ILC3), which then increases the level of IL-22 secretion, thus promoting the proliferation of intestinal epithelial cells and the progression of CRC.	Coker et al., 2019; Zhu et al., 2021
	Breast tumor	Induce Tregs and result in dysregulation of cytokine network and thereby facilitate tumor growth.	Ahmadi et al., 2019
	Liver cancer	Reprogramm tumor cell metabolism and contributes to the cancer progression dependent on NLRP6.	Liu et al., 2022

CaADH1, *Candida albicans* alcohol dehydrogenase 1; OSCC, oral squamous cell carcinoma; ILC3, intestinal innate lymphocytes 3.

### *Candida albicans* and gastric cancer

Gastric cancer (GC) is the fifth most common cancer in the world and the third most common cause of cancer-related death (Smyth et al., 2020). After the continuous development of high-throughput sequencing technology, research on the correlation between gastric microbiome (other than *Helicobacter pylori*) and GC has gradually emerged. A study described the composition and ecological changes of fungi by analyzing the metagenomic sequences in cancer foci and adjacent non-cancer tissues of 45 GC patients. The results showed that GC related fungal biological community was unbalanced, which was characterized by changes in fungal composition and ecology, and suggested that *C. albicans* might be used as a fungal biomarker of GC. With the significant increase of *C. albicans* in GC, the abundance of *Fusicolla acetilerea*, *Arcopilus aureus*, and *Fusicolla aquaeductuum* were increased, while *Candida glabrata*, *Aspergillus montevidensis*, *Saitozyma podzolica*, and *Penicillium arenicola* were obviously decreased (Zhong et al., 2021). In addition, *C. albicans* reduced the diversity and abundance of fungi in the stomach, thus enhancing the development of GC (Zhong et al., 2021; Table 1). However, these studies did not further clarify the specific molecular mechanism by which *C. albicans* is involved in the progression of GC.

### *Candida albicans* and colorectal cancer

Colorectal cancer (CRC) is the third most common cancer in the world and the second most common cause of cancer-related death (Siegel et al., 2021, 2022). It is well known that gut microbiota play critical roles in CRC development (Qing et al., 2022). Through intestinal microbiota biodiversity analysis, a study showed that fungal dysbiosis and altered fungal network might play an important role in the pathogenesis of CRC (Gao et al., 2017). Subsequently, another study reached the similar conclusion through the ecological analysis of gut microbiota (Coker et al., 2019). However, these studies did not clarify the specific role of symbiotic fungi in CRC. Recently, in fungal specific pattern recognition receptor *Dectin-3* knockout mice (*Dectin-3^–/–^* mice), the deletion of *Dectin-3* gene could lead to a significant increase in CRC development, and the fungal load in *Dectin-3^–/–^* mouse feces is significantly higher than that in wild-type (WT) mice. Interestingly, the proportion and abundance of *C. albicans* were significantly increased. The fecal fungus transplantation experiment further confirmed that the feces of *Dectin-3^–/–^* tumor bearing mice and *C. albicans* can promote the malignant process of CRC, and antifungal treatment can effectively alleviate the tumor load of *Dectin-3^–/–^* mice. *In vivo* and *in vitro* experiments also confirmed that *Dectin-3* gene deletion can damage the ability of macrophages to scavenge *C. albicans* and increase the load of fungi. These studies reveal the molecular mechanism of *C. albicans* in regulating intestinal immunity and promoting the development of CRC (Zhu et al., 2021; Table 1).

### *Candida albicans* and other cancers

It is well known that *Aspergillus flavus*, which produces aflatoxin, is closely related to liver cancer (Cai et al., 2020; Sun et al., 2021). *C. albicans* was also found to be closely related to liver cancer. A study found that the diversity of intestinal fungal community in patients with liver cancer decreased significantly, and the abundance of *C. albicans* increased. Abnormal colonization of *C. albicans* increased the size and weight of liver cancer, affected the cancer cell metabolism, thus promoting the progression of liver cancer dependent on nucleotide oligomerization domain-like receptor family pyrin domain containing 6 (NLRP6) (Liu et al., 2022). It indicates the harmful effect of *C. albicans* on liver cancer may be mediated by NLRP6, which provides a new target for the cancer treatment. Nevertheless, they have not identified the cell surface receptors that recognize *C. albicans* that can further activate NLRP6. Recently, another study showed that compared to the uninfected control group, *C. albicans* infection increased the number of Tregs in TME. In addition, compared to the tumor group, *C. albicans* infection increased tumor growth in the tumor/*Candida* infection group. It further shows that systemic infection of *C. albicans* could not only induce Tregs, but also lead to the imbalance of cytokine network, so as to promote the growth of tumor (Ahmadi et al., 2019; Table 1). Tregs have been shown to enhance tumor progression by inhibiting antitumor immune response (Deng et al., 2013; Lainé et al., 2021). However, the specific mechanism by which *C. albicans* promotes occurrence and development of breast cancer remains unknown. The fungal community of pancreatic ductal adenocarcinoma (PDA) was shown to be different from that of healthy controls. Furthermore, the fungal community of PDA was significantly enriched in *Malassezia*, which promoted tumor growth, while *Candida* could not accelerate tumor growth (Aykut et al., 2019). It indicates that *C. albicans* may not be involved in the progression of PDA. The role of fungal microorganisms in cancer development are gradually appreciated.

### *Candida albicans* and other microorganisms during cancer development

*Candida albicans* has synergy, symbiosis and antagonism with other microorganisms, which determines the role of the microbiota in which *C. albicans* is located. For example, the interaction of multiple microorganisms can improve the ability of *C. albicans* biofilm formation (Lohse et al., 2018), and then enhance the ability of *C. albicans* to invade the host. A study showed that the interaction between *C. albicans* and oral microorganisms might promote oral carcinogenesis (Arzmi et al., 2019). The metabolites of the polymicrobial membrane formed by *C. albicans*, *Actinomyces naeslundii* and *Streptococcus mutans* regulate the phenotype of cancer cells by increasing the adhesion of OSCC to extracellular matrix (ECM) and enhancing the expression of proinflammatory cytokines (Arzmi et al., 2019). However, *C. albicans* and *S. mutans* have antagonistic effects. *S. mutans* can inhibit the mycelial growth and biofilm formation of *C. albicans* and reduce its pathogenicity (Barbosa et al., 2016). Previous studies showed that *C. albicans* could inhibit *S. mutans* from producing extracellular polymeric substances (EPSs) and reduce its biofilm toxicity (Sztajer et al., 2014). It is also reported that oral actinomycetes (including *A. naeslundii*) also inhibit the proliferation, adhesion, metabolic enzyme activity, mycelial growth and biofilm formation of *C. albicans* (Guo et al., 2015). All these complicated interactions between multiple microorganisms may also explain the reduction of colonization on the surface of multiple microbial membranes and the differential regulation of oral cancer cell phenotypes, but not in single *C. albicans* and *S. mutans*.

## Effect of cancer and anticancer therapy on *Candida albicans*

In cancer, microbial communities in cancer-related areas usually change, including fungal communities. A study investigated the steady-state changes of microbial community during the occurrence of GC, and found that GC was significantly related to the changes of fungal community in the stomach, including decreased biodiversity and richness the increased proportion of opportunistic fungi (Yang et al., 2022). Another study also showed that the abundance of *C. albicans* increased in GC and *C. albicans* promoted cancer progression by reducing the diversity of fungi in the stomach (Zhong et al., 2021). In CRC, there was also an imbalance of fungal community, with increased abundance of *C. albicans* (Wang et al., 2021a). *C. albicans* may promote the development of CRC through Dectin-1/Wnt pathway (Wang et al., 2021a). These studies suggest that cancer may promote the proliferation of *C. albicans* in fungal communities, and *C. albicans* may also promote the development of cancer. Unfortunately, the mechanism of the interaction between cancer and fungi are still largely unclear.

On the one hand, when cancer occurs, the mucosal barrier function of the host is often damaged, which may cause the invasion of the conditional pathogen *C. albicans*. On the other hand, cancer may suppress host immune function, which promotes invasion of *C. albicans*. Finally, anticancer treatment may damage the host immunity and further increase the infection of *C. albicans*. However, some studies also show that anticancer drugs can inhibit the formation of *C. albicans* biofilm and reduce its invasiveness (Wakharde et al., 2018). Another study showed that anticancer drugs and radiation could enhance the virulence of *C. albicans* and increase the risk of systemic candidiasis (Ueta et al., 2001). In addition, with the extension of anticancer treatment duration, *C. albicans* may produce a large number of phospholipases to enhance its invasion (Ramla et al., 2016). So far, these studies have not clearly clarified the specific mechanism of anticancer treatment on the invasiveness of *C. albicans*.

## Possible carcinogenic mechanism of *Candida albicans*

Current studies suggest that *C. albicans* may promote the development of cancer through various mechanisms such as damaging the mucosal epithelium, producing carcinogens, triggering chronic inflammation, and inducing Th17 immune responses. However, some of these mechanisms still lack strong and direct evidence and need to be further verified.

### *Candida albicans* damages epithelial mucosal cells and invades the host

Epithelial mucosal cells are the first line of defense for the host protection against the invasion of pathogenic microorganisms. Adhesion of *C. albicans* to epithelial cells is the first step of fungal colonization and invasion. Subsequently, it invades epithelial cells by inducing endocytosis and active infiltration, which is a key step in the pathogenesis of *Candida* (Maza et al., 2017; Allert et al., 2018; Lipke, 2018). Among them, *C. albicans* invasive enzymes destroy the integrity of mucosal tissue structure and enhance its virulence; hemolytic factors help them obtain nutrients for its survival and reproduction; phenotypic transformation can help *C. albicans* adapt to the host tissue environment; adhesin can assist in its colonization and invasion of host cells (Zhu and Filler, 2010; Tao et al., 2014; Furlaneto et al., 2015; Noble et al., 2017). Once *C. albicans* invades epithelial mucosal cells, it may induce apoptosis and necrosis, destroy the host epithelial defense barrier, and finally leading to the structural changes of epithelial cells (Richardson et al., 2018; Mogavero and Hube, 2021). These are the preconditions for the cancer promoting *C. albicans* infection. Epithelial cells are damaged and the normal structure is changed, which results in abnormal proliferation and the formation of oral cancer (Engku Nasrullah Satiman et al., 2020). There is a significant positive correlation between *C. albicans* infection and oral mucosal epithelial dysplasia, and the deterioration of epithelial dysplasia induced by *C. albicans* infection occurs (Barrett et al., 1998). Multiple studies showed that *C. albicans* could induce epithelial dysplasia and further malignant tumor (McCullough et al., 2002; Dwivedi et al., 2009).

### *Candida albicans* produces carcinogens

The substances such as nitrosamine (Mohd Bakri et al., 2010) and acetaldehyde (Stornetta et al., 2018) produced by *C. albicans* play a certain role in promoting cancer development. A study showed that *C. albicans* had higher nitrosation production potential than other fungi, and could convert N-benzylmethylamine (BMA) in vegetables, herring oil and freeze-dried coffee and nitrite produced by other microorganisms in the host’s mouth into N-nitroso-benzylmethylamine (NBMA), thus inducing the occurrence and development of OSCC (Krogh, 1990). However, the mechanism of direct carcinogenesis is still controversial. It may also due to the fact that the integrity of oral mucosal cell barrier and smoking and other risk factors enhance the virulence of *C. albicans* and jointly promote oral carcinogenesis (Ye et al., 2021). Recently, a study compared the ability of *Candida* isolated from oral cancer patients and matched oral healthy subjects to produce acetaldehyde. The results showed that *Candida* strains producing a large amount of acetaldehyde were more in patients with oral cancer than in healthy volunteers, further indicating the possible role of *Candida* in enhancing the occurrence of oral cancer (Alnuaimi et al., 2016). *C. albicans* may secrete alcohol dehydrogenase, which converts ethanol into acetaldehyde and participates in the carcinogenic process (Stornetta et al., 2018). Acetaldehyde can induce DNA adducts to interfere with DNA replication, resulting in point mutation and chromosome aberration. At the same time, it affects the enzymes involved in cytosine methylation and DNA repair, leading to protooncogene activation and cell cycle disorder, which may lead to tumor progression. In addition, acetaldehyde can also cause mitochondrial damage and promote apoptosis, which can be activated by NF-κB to offset. For example, in GC, NF-κB induced IL-6 upregulates the antiapoptotic gene MCL1 to inhibit DNA repair and apoptosis (Lin et al., 2001; Wang et al., 2021b). The combination of acetaldehyde and glutathione indirectly increases the production of reactive oxygen species (ROS), thus inducing DNA damage, which is conducive to the progress of cancer (Ramirez-Garcia et al., 2016; Mizumoto et al., 2017; Johnson et al., 2021). However, the correlation between the acetaldehyde concentration produced by *C. albicans* and the severity of cancer tissue is unknown, and the mechanism to promote cancer development is still unclear.

### *Candida albicans* induces tumor microenvironment

Stromal cells are composed of fibroblasts, vascular cells and inflammatory immune cells, which together constitute the TME. Both chronic disease induced inflammation and tumor induced inflammation have a great impact on the composition of TME, especially on the plasticity of tumor and stromal cells. Inflammatory substances released by immune cells in TME can directly affect precancerous cells and cancer cells by increasing the cell proliferation and their resistance to cell death and stress, so as to directly promote tumor progression (Greten and Grivennikov, 2019). Therefore, chronic inflammatory response plays an important role in the occurrence and development of tumors. Persistent *Candida* epithelial mucosal colonization and infection can also cause a chronic inflammatory state. *C. albicans* recognizes Toll like receptors (TLRs) and C-type lectin-like receptors (CLRs), and then activates the corresponding MAPK and NF-κB. Interferon and inflammatory signaling pathway promote the expression of a variety of related inflammatory genes and play a role in the connection between benign and malignant diseases. Studies have found that the expression of inflammatory factors prostaglandin E2 (PGE2) and MMPs (mainly MMP-9) increased after *C. albicans* infection in oral epithelial cells (Claveau et al., 2004; Nasry et al., 2018). In addition, prostaglandins, cyclooxygenase (COX) enzymes and MMPs can inhibit tumor suppressor genes through DNA methylation and post-translational modification, leading to the occurrence and development of cancer (Munn, 2017).

Studies have shown that PGE2 are overexpressed in a variety of cancer types, including breast cancer (Nandi et al., 2017), oral cancer (Tao et al., 2021), and CRC (Karpisheh et al., 2019). However, *C. albicans* can induce human peripheral blood mononuclear cells (PBMC) to produce PGE2 (Smeekens et al., 2010). PGE2 promotes tumorigenesis by producing ROS, stimulating carcinogenic transcription factors, inhibiting antitumor immune response (Liu et al., 2015), and enhancing angiogenesis (Mizuno et al., 2019). PGE2 inhibits the cytotoxic function of NK cells and prevents them from producing IFN-γ, and promotes malignant growth by avoiding type I interferon and T cell-mediated cell death. PGE2 promotes the inhibitory activity of Tregs, and contributes to the maturation of Tregs, thereby inhibiting antitumor immunity (Nasry et al., 2018). In addition, PGE2 increases MDSCs, and inhibits innate and adaptive antitumor immunity by downregulating cytokines of macrophages, inhibiting cytotoxicity of NK cells, blocking activation of cytotoxic T cells, and regulating the development of Tregs. PGE2 facilitates bone marrow mesenchymal stem cells migrate into the tumor environment and enables malignant cells to proliferate without interference from the host immune system (Nasry et al., 2018). PGE2 binds to PGE receptor (EP1) and activates protein kinase δ (protein kinase Cδ, PKCδ)/c-Src/AP-1 signaling pathway, upregulates intercellular adhesion molecule 1 (ICAM-1), and promotes the metastasis of cancer cells (Yang et al., 2010). However, the exact mechanism by which PGE2 produced by *C. albicans* in the process of chronic inflammation promotes cancer development is not clear.

Other studies have shown that MMP-9 is highly expressed in cancer tissues such as oral cancer (Xie et al., 2020), GC (Dong et al., 2020), CRC (Guo et al., 2020), breast cancer (Nazir et al., 2019), and cervical cancer (Azevedo Martins et al., 2020), and has been used as a potential marker of cancers (Huang, 2018). Some studies have shown that the high expression of MMP-9 leads to the enhancement of tumor invasion, which maybe because the effect on the transforming growth factor (TGF-β1) induced epithelial mesenchymal transition (EMT) process, which promotes the invasion and metastasis of cancer (Li et al., 2020a). At the same time, MMP-9 can degrade type IV collagen of basement membrane, destroy the integrity of basement membrane, and also contribute to the invasion and metastasis of tumor cells (Koontongkaew, 2013). In addition, MMP-9 degrades ECM components and activates angiogenic factor VEGF and TGF-β helps cancer angiogenesis, and cleavage of osteopontin (OPN) also contributes to cancer metastasis (Quintero-Fabián et al., 2019). These results suggest that MMP-9 has a role of cancer promoting mechanism. However, a study showed that epithelial origin MMP-9 exerts tumor inhibitory effect by activating MMP9-Notch1-ARF-p53 axis, resulting in increased apoptosis, and starts cell cycle arrest by activating p21WAF1/Cip1, and checks the damaged DNA until DNA repair. In addition, in colitis associated CRC, MMP-9 can prevent γH2AX reduced levels of genotoxicity, also plays a tumor suppressive role (Walter et al., 2017). Subsequently, another study found that the expression of MMP-9 was related to the decrease of ROS level, the decrease of DNA damage and the upregulation of mismatch repair pathway. This suggests that the expression of MMP-9 is a natural biological way to inhibit CRC by limiting ROS accumulation and colonic DNA damage. Therefore, inhibition of MMP-9 may be harmful to patients with CRC (Walter et al., 2020). These results indicate that MMP-9 has a protective host effect in CRC. Therefore, MMP-9 has both cancer promoting and inhibiting cancer effects. However, it is not clear whether MMP-9 produced by host with *C. albicans* infection plays a role in promoting or inhibiting cancer formation. Whether it is related to the site of *C. albicans* infection or the type of tumor formation has not been reported. These need to be further studied.

*Candida albicans* infection induces both host innate and adaptive immune responses, and the core of protection is often from adaptive Th1 and Th17 cell immune response, which is also considered to be the primary factor in the successful immune defense against *C. albicans* infection (Chen and Kolls, 2017). However, Th17 cells are found in various types of human tumors. Th17 cells and their effector molecules (such as IL-17 and IL-22) can regulate oncogene activated cancer cells themselves and adjacent normal epithelial cells, fibroblasts, endothelial cells, and other stromal cells (Chang, 2019). Current studies have found that IL-17 could promote tumor growth through IL-6-Stat3 signaling pathway (Li et al., 2020b), and also release IFN-γ and other cytokines through stimulating T cells, dendritic cells, NK cells and other immune cells, thus inhibiting tumor growth (Kryczek et al., 2009; Martin-Orozco et al., 2009). In addition to its direct effect on tumor, IL-17 can also reshape the TME by recruiting neutrophils and macrophages and promoting tumor occurrence, development, and metastasis (Rei et al., 2014; Liang and Ferrara, 2016). IL-17 driven antitumor immunity is attributed to its ability to recruit dendritic cells (You et al., 2018). These may be related to various tumor types or TME. Recently, some studies have found that *C. albicans* can induce the increase of glycolysis of macrophages through HIF-1 pathway and promote the secretion and release of IL-17 by macrophages. Increased IL-17 can effectively promote the expression of STAT3 and AHR transcription factors in intestinal innate lymphocyte 3 (ILC3), which in turn leads to the increase of IL-22 and promotes the proliferation of intestinal epithelial cells and the progress of CRC (Zhu et al., 2021). However, the exact mechanism of the immune response induced by *C. albicans* infection remains unclear.

## Conclusion

At present, most of studies on interaction between *C. albicans* and cancer are epidemiological survey or descriptive studies. There are few molecular mechanistic studies in this field. Early researchers simply believe that cancer patients are prone to *Candida* infection. However, tumor is a disease caused by multiple factors. In the mouse model of oral carcinogenesis, a study showed that infection with *C. albicans* alone could not lead to oral cell dysplasia or OSCC, which requires pretreatment with epithelial carcinogenesis inducer (Dwivedi et al., 2009). *C. albicans* can promote the occurrence and development of cancer to a certain extent, which may be the result of synergy with the host’s own state and other factors. For example, defective host immunity provides opportunities for *C. albicans* infection; long-term smoking and drinking provide conditions for *C. albicans* to produce carcinogenic metabolites; chronic inflammation provides TME and other common factors for *C. albicans* to promote cancer, and promotes the occurrence and development of cancers.

In conclusion, various adverse factors cause compromised host immunity, leading to *C. albicans* infection. *C. albicans* infection increases the risk of cancer development and exacerbates cancer progression. Recent studies have shown that *C. albicans* infection may participate in the progression of cancer by damaging the epithelial mucosal barrier, producing carcinogenic metabolites, inducing chronic inflammation and Th17 immune response. The progression of cancer further aggravates *C. albicans* infection. The two promote each other and aggravate the malignant process of cancer development. Therefore, it seems that *C. albicans* infection may be accompanied by cancer development, and the two promote each other, which in turn aggravates the process of malignancy. It is hoped that these can provide direction for the study of the correlation between *C. albicans* and cancers, and also provide new ideas for the prevention, diagnosis, and treatment of cancers.

## Author contributions

ZL: original idea, planning, and editing. DY: writing and editing. Both authors read and agreed to the published version of the manuscript.
